# Supplementary material for: Identification of Cyanobacterial Strains with Potential for the Treatment of Obesity-Related Co-Morbidities by Bioactivity, Toxicity Evaluation and Metabolite Profiling
Source: Mar Drugs. 2019 May 10;17(5):280. doi: 10.3390/md17050280 (PMC6562398; doi:10.3390/md17050280)
Supplement: Supplementary file 1 [file marinedrugs-17-00280-s001.pdf]

Identification of cyanobacterial strains with potential for the treatment of obesity-related co-morbidities by bioactivity, toxicity evaluation and metabolite profiling

Margarida Costa [2], Filipa Rosa [1], Tiago Ribeiro [1], Rene Bautista [3], Marco Bonaldo [5], Natália Gonçalves Silva [1], Finnur Eyriksson [4], Margrét Thorsteinsdóttir [2], Siegfried Ussar [3], Ralph Urbatzka [1*]

[1] Interdisciplinary Centre of Marine and Environmental Research (CIIMAR/CIMAR), University of Porto, Avenida General Norton de Matos, s/n, 4450-208 Matosinhos, Portugal
[2] Faculty of Pharmaceutical Sciences, University of Iceland, Hofsvallagata 53, 107 Reykjavik, Iceland;
[3] HMGU, Helmholtz Centre Munich, Germany
[4] ArcticMass, Sturlugata 8, 101 Reykjavik, Iceland
[5] INBB, Consorzio Interuniversitario Biosistemi e Biostrutture, Rome, Italy
* Correspondent author: Ralph Urbatzka; rurbatzka@ciimar.up.pt, Tel.: 00351 – 223 401 818

Table of content **Pages**

Table 1 – List of cyanobacterial strains 2-4

Table 2 - List of qPCR primers used in bioactivity screening. 5

Figures 1 to 12 – PCA plots of cyanobacterial fractions A - I. 5-17

Figure 13 – Bright field images of brown adipocytes 18

Tables 3 to 6 - Identification of the compounds present on each fraction. 19-23

Table 1 - List of cyanobacterial strains. The corresponding taxon, growth environment, sample location, related publications, protocol of fractionation and sample codes are shown. More information can be found in [14]. IPE, increased polarity extraction; VLC, vacuum liquid chromatography. All locations in Portugal, if not indicated otherwise.

	Strain code	Environment	Sampling location	Fractionation/Extraction	Sample Codes
Planktothrix mougeotii	LEGE 07230	Aquatic, freshwater, floating masses, from a secondary decanter tank bank	Vila Nova de Gaia	VLC	1 - 9
***Tychonema* sp.**	LEGE 07196	Aquatic, freshwater, biofilm, from a biological treatment tank outlet	Vila Nova de Gaia	VLC	10 – 18
***Nodosilinea* sp.**	LEGE 06001	Aquatic, marine, sea water sample, coastal, surf zone	Praia de Buarcos	VLC	19 – 27
***Synechocystis* sp.**	LEGE 06079	Aquatic, brackish, mesotidal zone, benthic (freshwater)	Douro estuary, Vila Nova de Gaia	VLC	28 – 35
Mycrocistis aeruginosa	LEGE 91094	Aquatic, freshwater, pond, water sample	Lagoa de Mira	VLC	36 – 44
Nodosilinea nodulosa	LEGE 07084	Aquatic, brackish, mesotidal zone, benthic (freshwater)	Minho estuary, Caminha	VLC	45 – 53
Nodosilinea nodulosa	LEGE 06104	Aquatic, marine, tide puddle, rock surface scraping	Praia da Luz, Lagos	VLC	54 – 62
Synechocystis sp.	LEGE 07211	Aquatic, marine, biofilm, from a biological treatment tank outlet	Vila Nova de Gaia	VLC	63 - 74
Unidentified filamentous Cyanobacterium	LEGE 07212	Aquatic, freshwater, biofilm, from a biological treatment tank outlet	Vila Nova de Gaia	VLC	75 – 84
***Tychonema* sp.**	LEGE 06363	Aquatic, freshwater, biofilm, from a biological treatment tank outlet	Vila Nova de Gaia	VLC	85 – 95
Limnothrix sp.	LEGE 00237	Aquatic, freshwater, water sample	Mortagua	VLC	96 – 105
Nodosilinea sp.	LEGE 03283	Aquatic, freshwater, water sample, dam reservoir	Maranhão	VLC	106 – 115
Chroococcidiopsis sp.	LEGE 06174	Aquatic, marine, sea water sample, coastal, surf zone	Praia da Aguda, Arcozelo	VLC	116 – 125
Cyanobium sp.	LEGE 07175	Aquatic, marine, sea water sample, coastal, surf zone	Praia do Martinhal, Vila do Bispo	VLC	126 – 137
**Unidentified filamentous Synechococcales**	LEGE 07075	Aquatic, brackish, mesotidal zone, benthic	Douro estuary, Porto	VLC	138 – 146
Synechococcus sp.	LEGE 11428	Aquatic, marine, subtidal sample,	'A Pedra', diving spot in	VLC	147 – 155

		epilithic (13m depth), about 200 m off the shore	front of the fort 'Castelo do Queijo'		
**Unidentified filamentous cyanobacterium**	LEGE 00060	Aquatic, freshwater	Morocco: Oued Mellah	VLC	156 -164
Cuspidothrix issatschenkoi	LEGE 00247	Aquatic, freshwater, water sample	Maranhão dam reservoir, Benavila	VLC	165 – 173
Dolichospermum sp.	LEGE 00246	Aquatic, freshwater, water sample	Maranhão dam reservoir, Benavila	VLC	174 – 182
Nodosilinea nodulosa	LEGE 06152	Aquatic, marina, sea water sample, coastal, surf zone	Praia de Lavadores, Canideo	IPE	183 – 185
Leptolyngbya cf. Halophilla	LEGE 06102	Aquatic, marine, tide pool, on a submerge stone	Praia de São Bartolomeu do Mar, Esposende	IPE	186 – 188
Leptolyngbya mycoidea	LEGE 06108	Aquatic, marine, tide puddle, rock surface scraping	Praia da Luz, Lagos	IPE	189 – 191
Leptolyngbya mycoidea	LEGE 06118	Aquatic, marine, tide puddle, rock surface scraping	Praia da Luz, Lagos	IPE	192 – 194
Leptolyngbya mycoidea	LEGE 06009	Aquatic, marine, intertidal zone, epilithic	Praia da Foz do Arelho, Caldas da Rainha	IPE	195 – 197
Leptolyngbya fragilis	LEGE 07167	Aquatic, marine, tide puddle, rock surface scraping	Praia de Lavadores, Canidelo	IPE	198 – 200
Pseudanabaena aff. Curta	LEGE 07160	Aquatic, marine, tide puddle, on a submerged stone	Praia de Olhos d'Água, Albufeira	IPE	201 – 203
Pseudanabaena aff.	LEGE 07169	Aquatic, marine, intertidal zone, wave-exposed rock	Praia da Aguda, Arcozelo	IPE	204 – 206
Pseudanabaena aff. Persicina	LEGE 07163	Aquatic, marine, intertidal zone, on a *Mytilus* sp. shell	Praia de Moledo, Caminha	IPE	207 – 209
**unidentified filamentous Synechococcales**	LEGE 06144	Aquatic, marine, intertidal zone, wave-sheltered zone, sand	Praia de Burgau, Budens	IPE	210 – 212
***Pseudanabaena* sp.**	LEGE 06194	-	-	IPE	213 – 215
Cyanobium sp.	LEGE 06098	Aquatic, marine, intertidal zone, on a green macroalgae	Praia do Martinhal, Vila do Bispo	IPE	216 – 218
Cyanobium sp.	LEGE 06134	Aquatic, marine, intertidal zone, *Sabellaria* sp. reef, epipsamic	Praia de Moledo, Caminha	IPE	219 – 221
Cyanobium sp.	LEGE 07186	Aquatic, marine, tide pool, on a submerged stone	Praia do Martinhal, Vila do Bispo	IPE	222 – 224
***Cyanobium* sp.**	LEGE 06113	Intertidal zone, *Sabellaria* sp. reef, epipsamic	Praia da Aguda, Arcozelo	IPE	225 – 227

***Cyanobium* sp.**	LEGE 06137	Aquatic, marine, intertidal zone, wave-exposed rock, surface scraping	Praia de Lavadores, Canidelo	IPE	228 – 230
***Cyanobium* sp.**	LEGE 06097	Aquatic, marine, intertidal zone, on a green macroalgae	Praia do Martinhal, Vila do Bispo	IPE	231 – 233
***Cyanobium* sp.**	LEGE 06139	Aquatic, marine, intertidal zone, on a *Mytilus* sp. shell	Praia da Aguda, Arcozelo	IPE	234 – 236
Synechococcus nidulans	LEGE 07171	Aquatic, marine, tide puddle, air-exposed rock surface scraping	Praia de Burgau, Budens	IPE	237 – 239
***Synechococcus* sp.**	LEGE 07172	Aquatic, marine, tide puddle, on a submerged stone	Praia de Olhos d'Água, Albufeira	IPE	240 – 242
***Synechococcus* sp.**	LEGE 06005	Aquatic, marine, sea water sample, coastal, surf zone	São Pedro de Moel	IPE	243 – 245
***Synechococcus* sp.**	LEGE 06026	Aquatic, marine, intertidal zone, wave-sheltered rock	Praia da Empa, Ericeira	IPE	246 – 248
Synechocystis salina	LEGE 06099	Aquatic, marine, wave-exposed tide pool, rock surface scraping	Praia de Moledo, Caminha	IPE	249 - 251
Synechocystis salina	LEGE 06155	Aquatic, marine, tide pool, rock surface scraping	Praia de São Bartolomeu do Mar, Esposende	IPE	252 – 254
Synechocystis salina	LEGE 07173	Aquatic, marine, tide puddle, on a submerged stone	Praia de Olhos d'Água, Albufeira	IPE	255 – 257
Romeria sp.	LEGE 06013	Aquatic, marine, intertidal zone, wave-exposed rock	Praia da Foz do Arelho, Caldas da Rainha	IPE	258 – 260
Romeria aff. Gracilis	LEGE 07310	Aquatic, brackish, mesotidal zone, benthic	Minho estuary, Caminha	IPE	261 – 263

Table 2 - List of qPCR primers used in bioactivity screening.

Gene	Forward primer (5´-3´)	Reverse primer (5´-3´)
UCP1	AGCCATCTGCATGGGATCAAA	GGGTCGTCCCTTTCCAAAGTG
PPARγ	CCCTGGCAAAGCATTTGTAT	GAAACTGGCACCCTTGAAAA
TBP	ACCCTTCACCAATGACTCCTATG	TGACTGCAGCAAATCGCTTGG

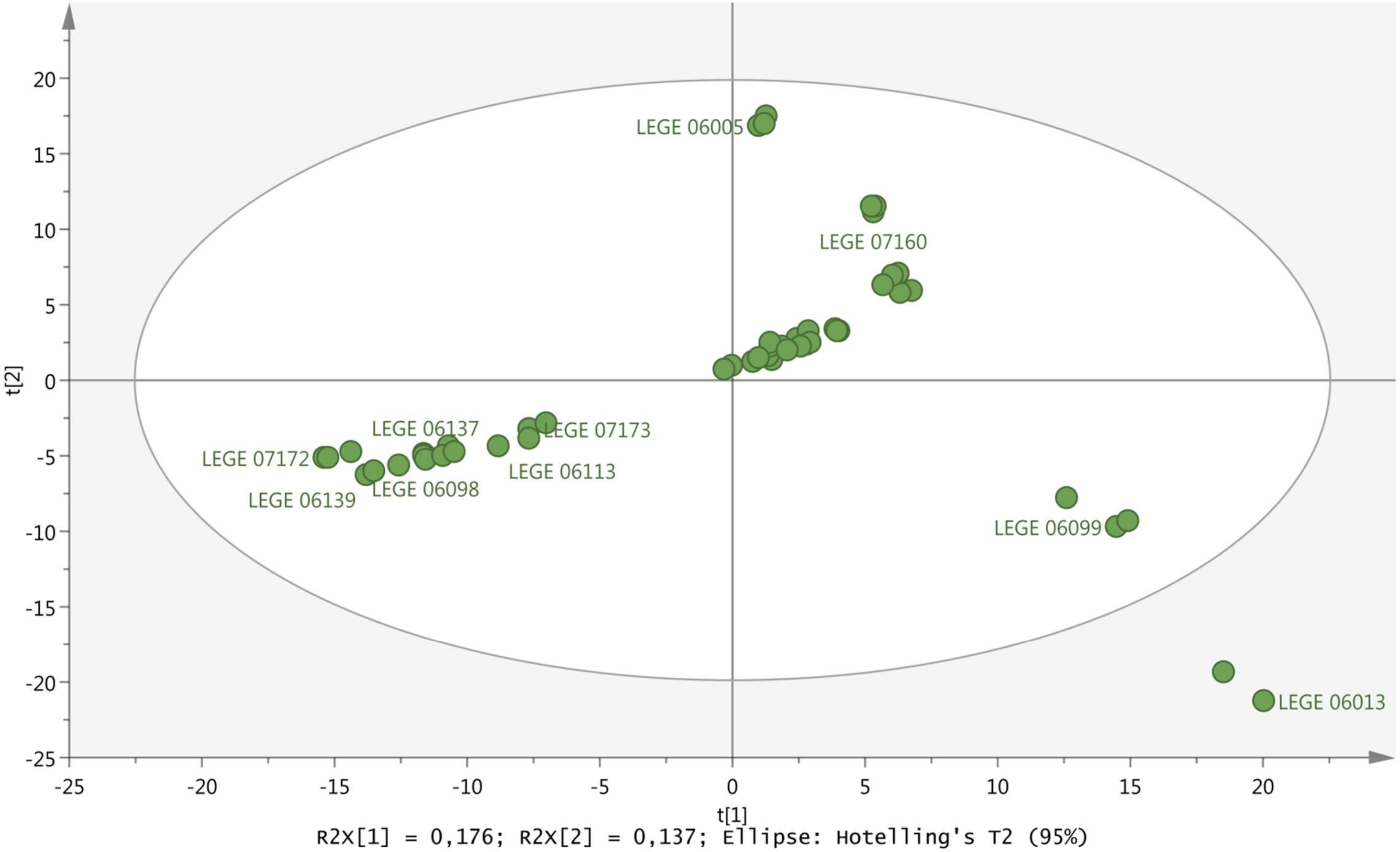

Figure 1 - Principal component analysis (scores plot) of cyanobacterial fractions A (IPE).

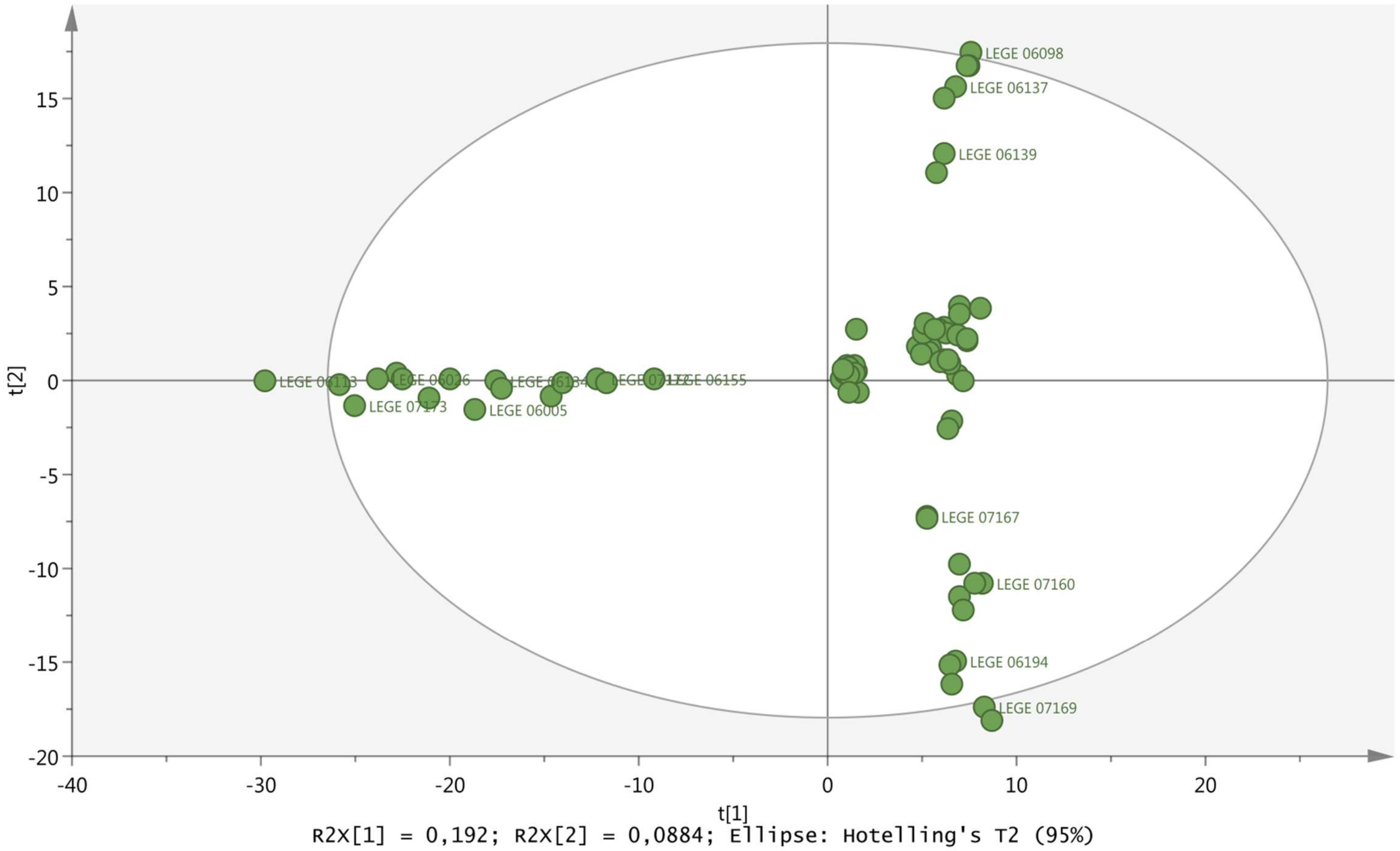

Figure 2 - Principal component analysis (scores plot) of cyanobacterial fractions B (IPE).

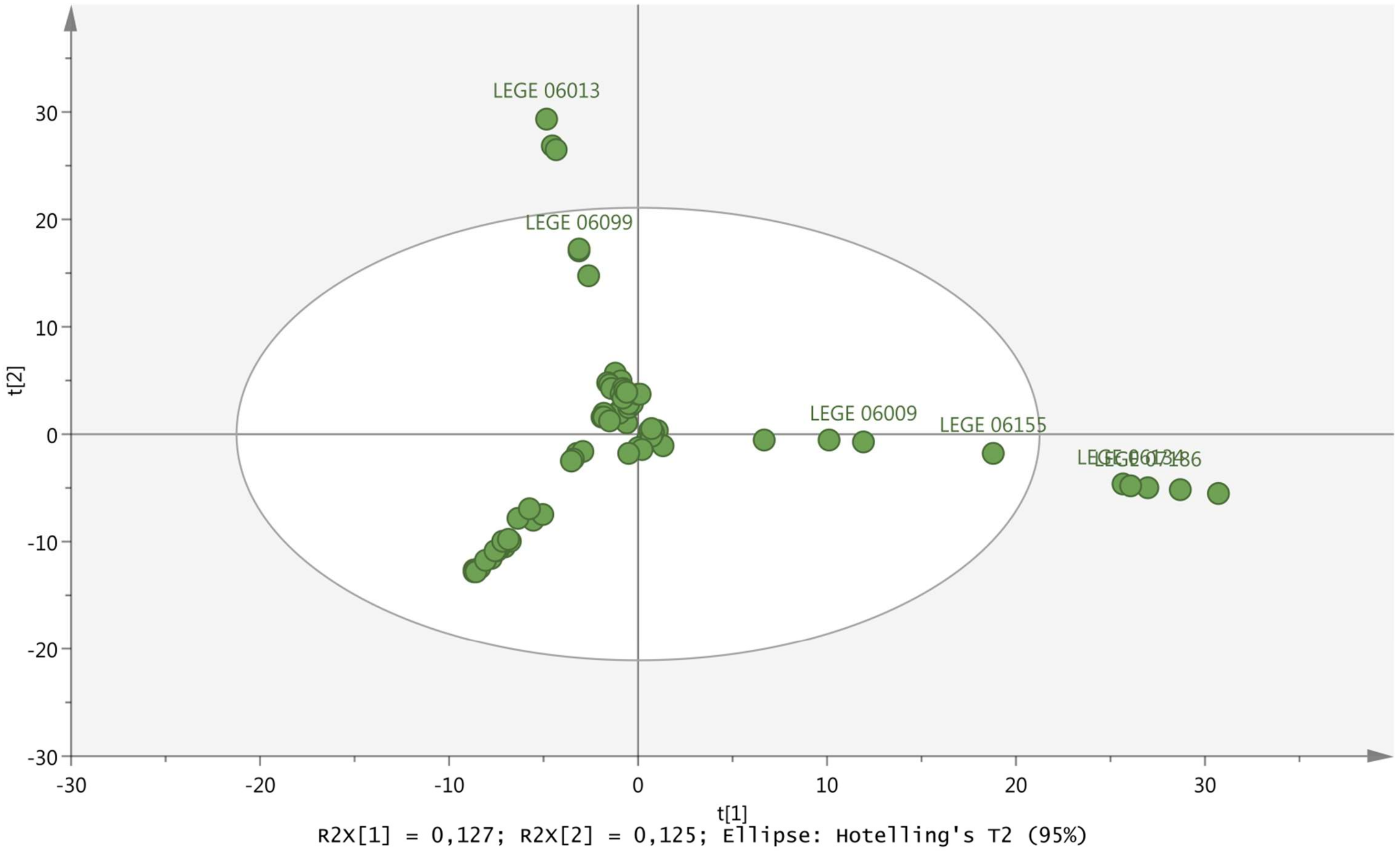

Figure 3 - Principal component analysis (scores plot) of cyanobacterial fractions C (IPE).

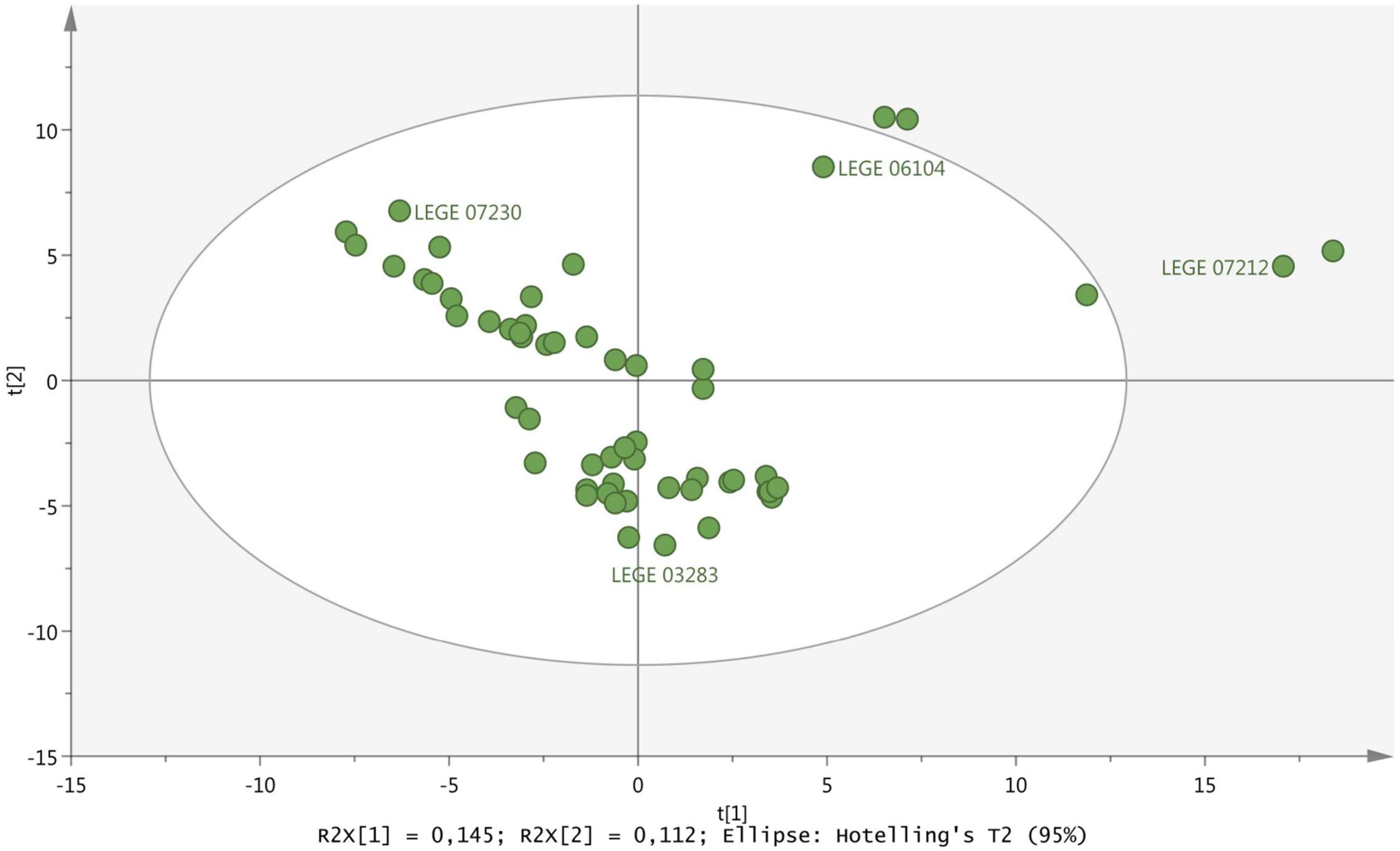

Figure 4 - Principal component analysis (scores plot) of cyanobacterial fractions A (VLC).

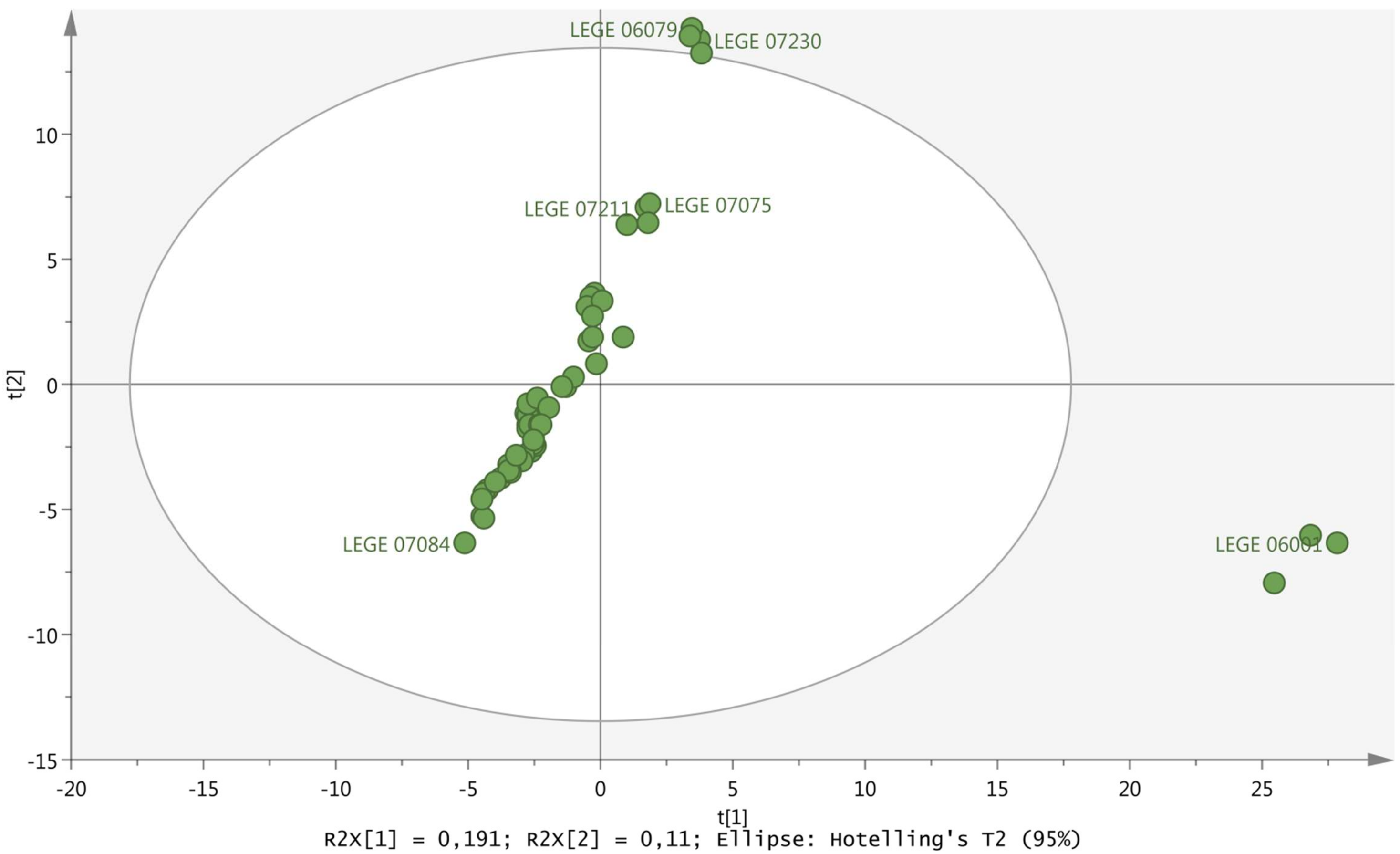

Figure 5 - Principal component analysis (scores plot) of cyanobacterial fractions B (VLC).

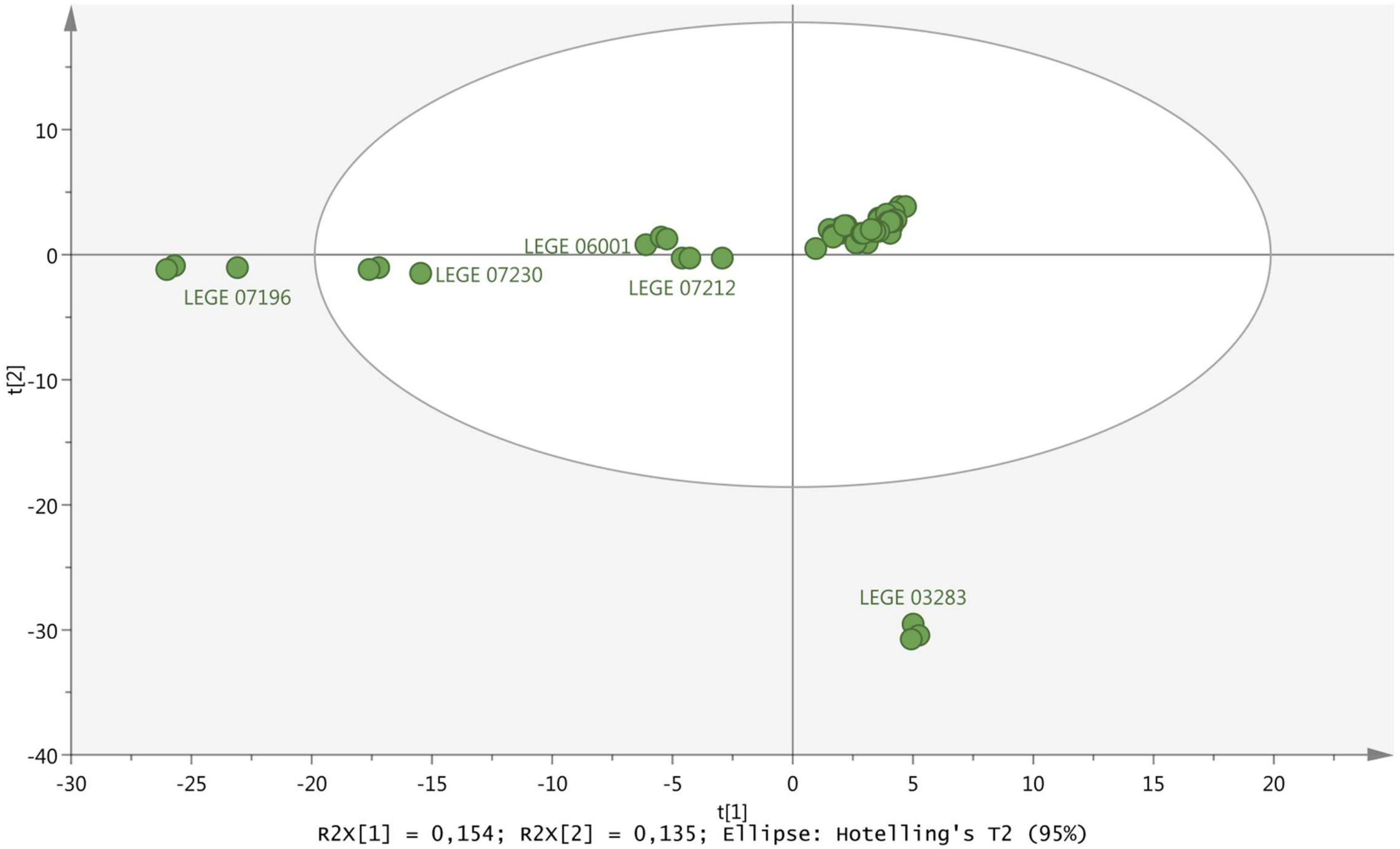

Figure 6 - Principal component analysis (scores plot) of cyanobacterial fractions C (VLC).

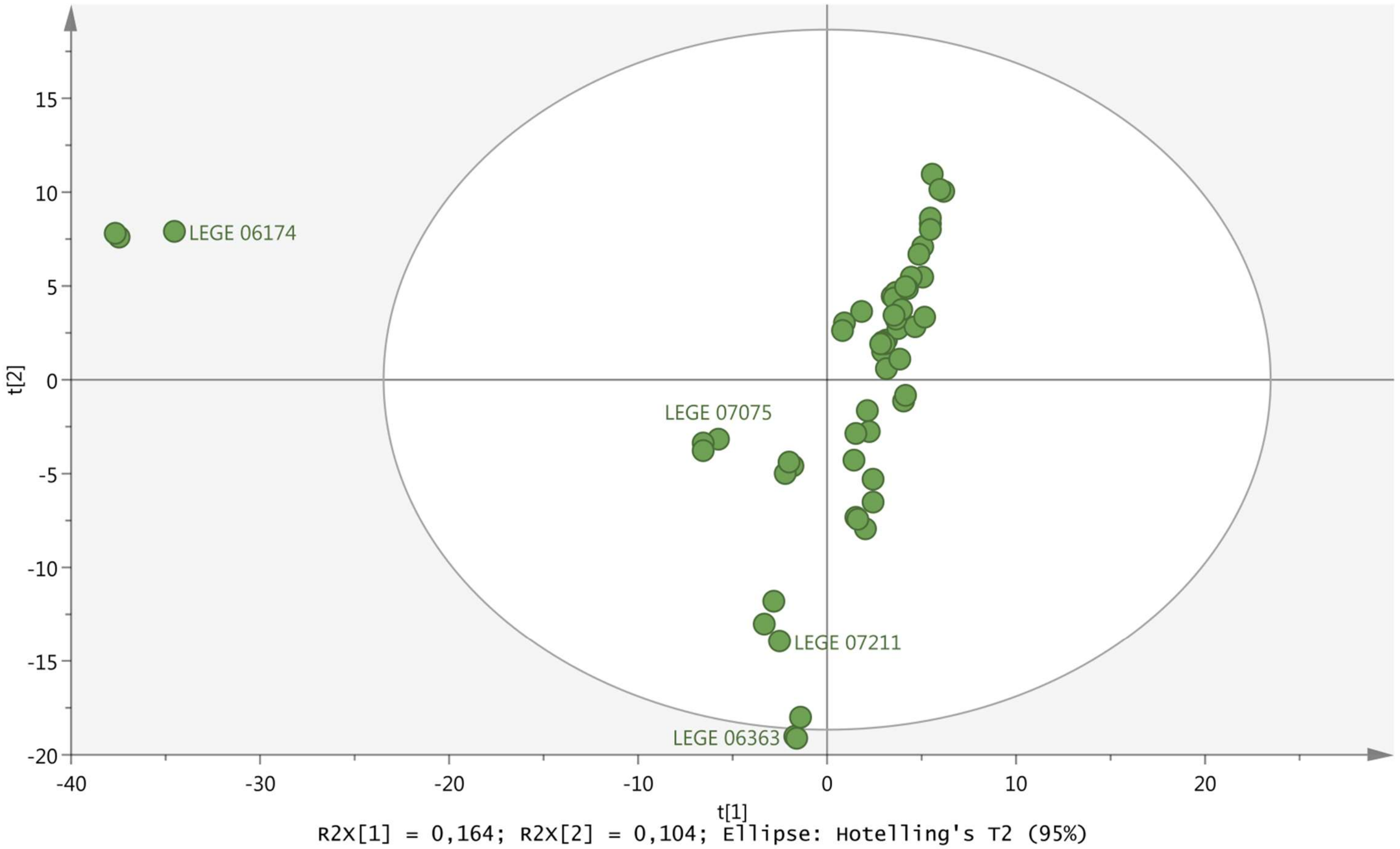

Figure 7 - Principal component analysis (scores plot) of cyanobacterial fractions D (VLC).

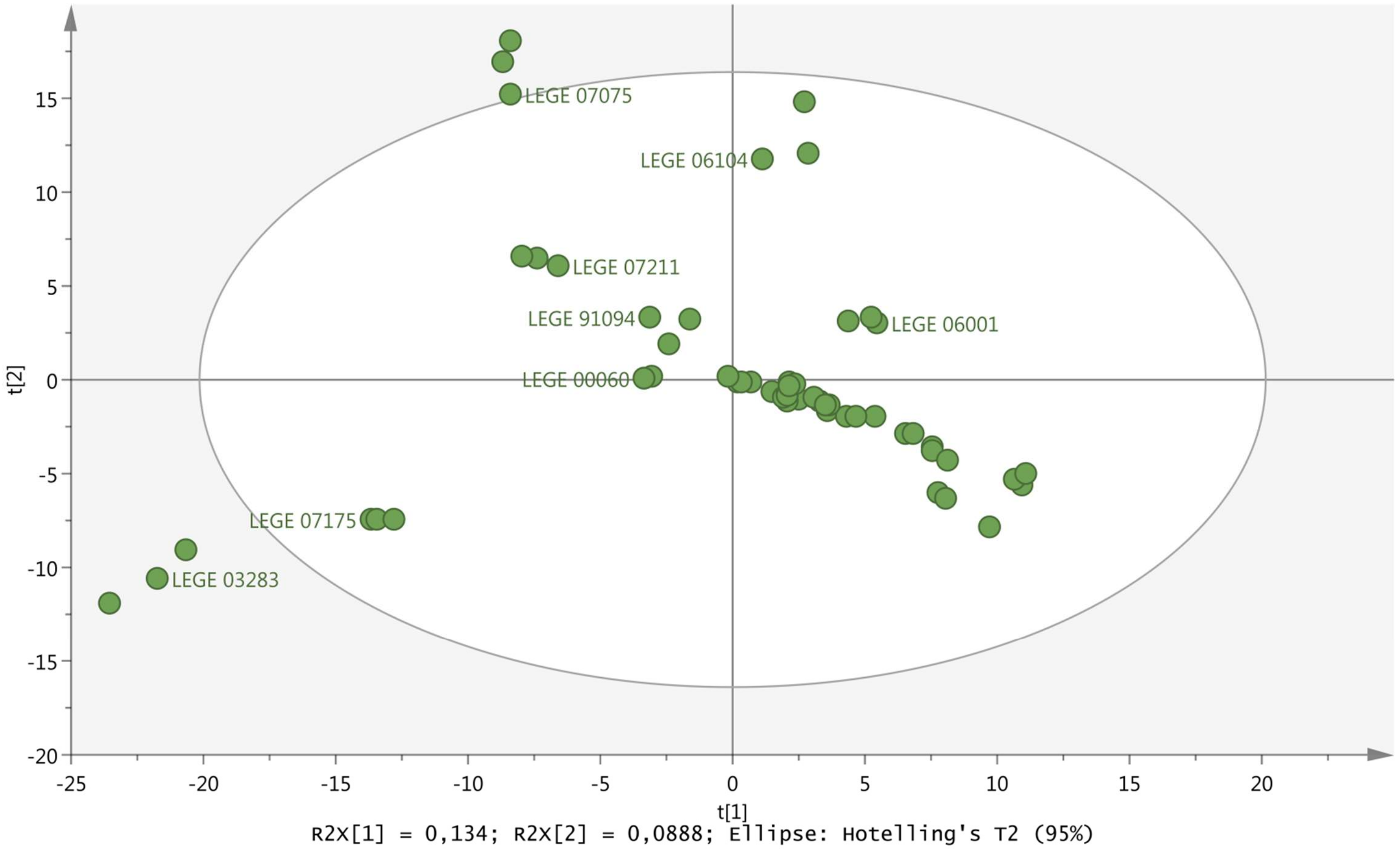

Figure 8 - Principal component analysis (scores plot) of cyanobacterial fractions E (VLC).

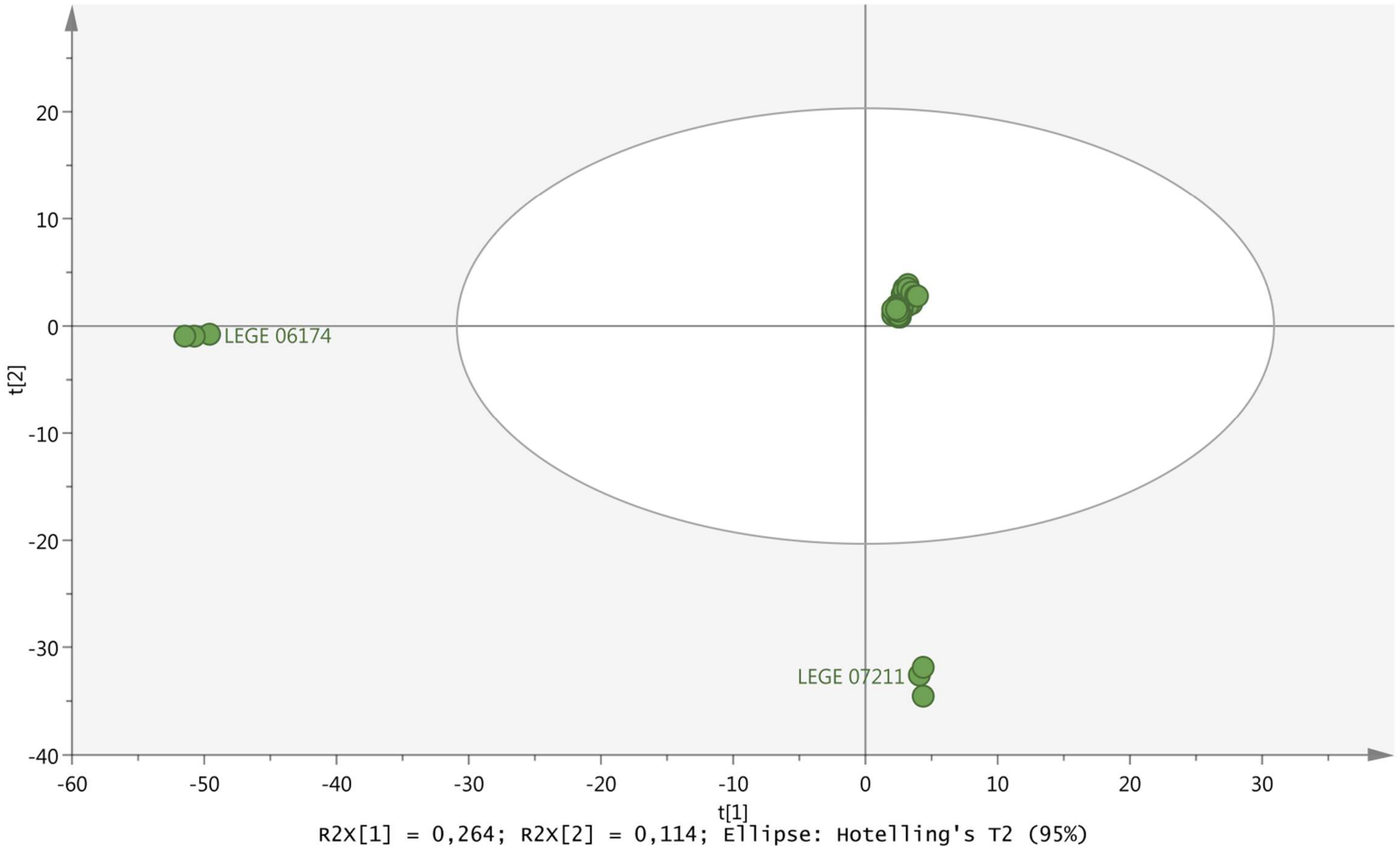

Figure 9 - Principal component analysis (scores plot) of cyanobacterial fractions F (VLC).

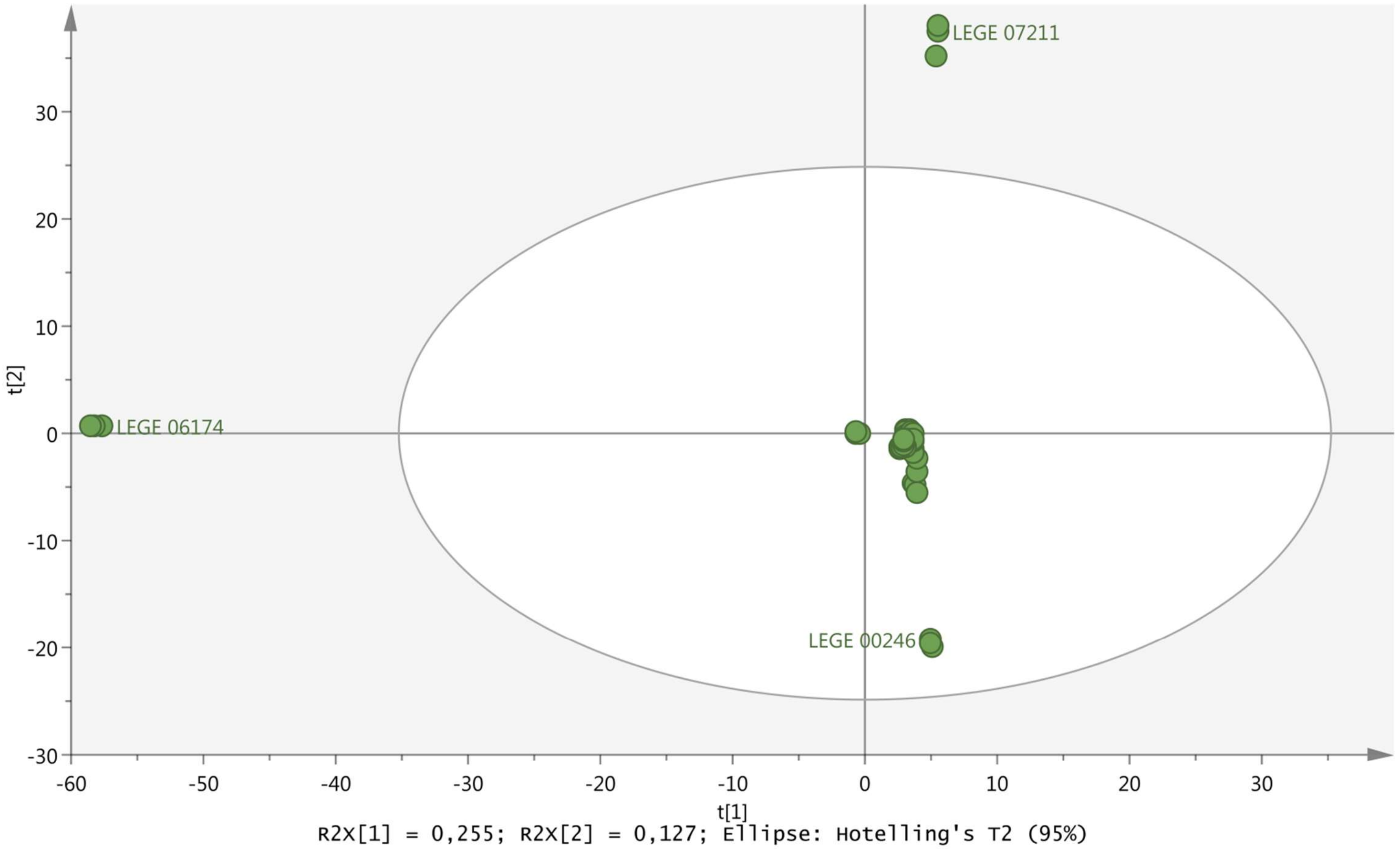

Figure 10 - Principal component analysis (scores plot) of cyanobacterial fractions G (VLC).

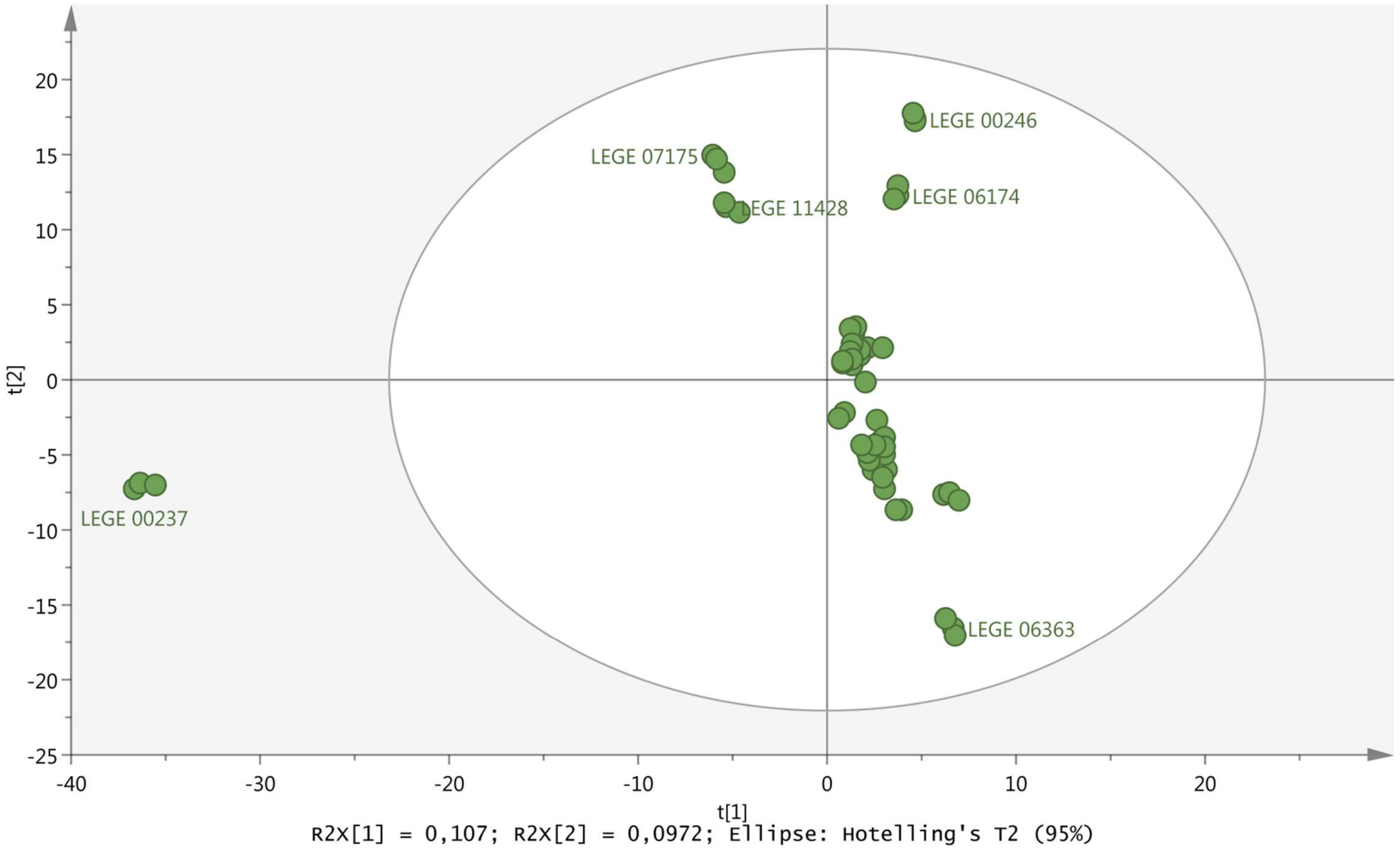

Figure 11 - Principal component analysis (scores plot) of cyanobacterial fractions H (VLC).

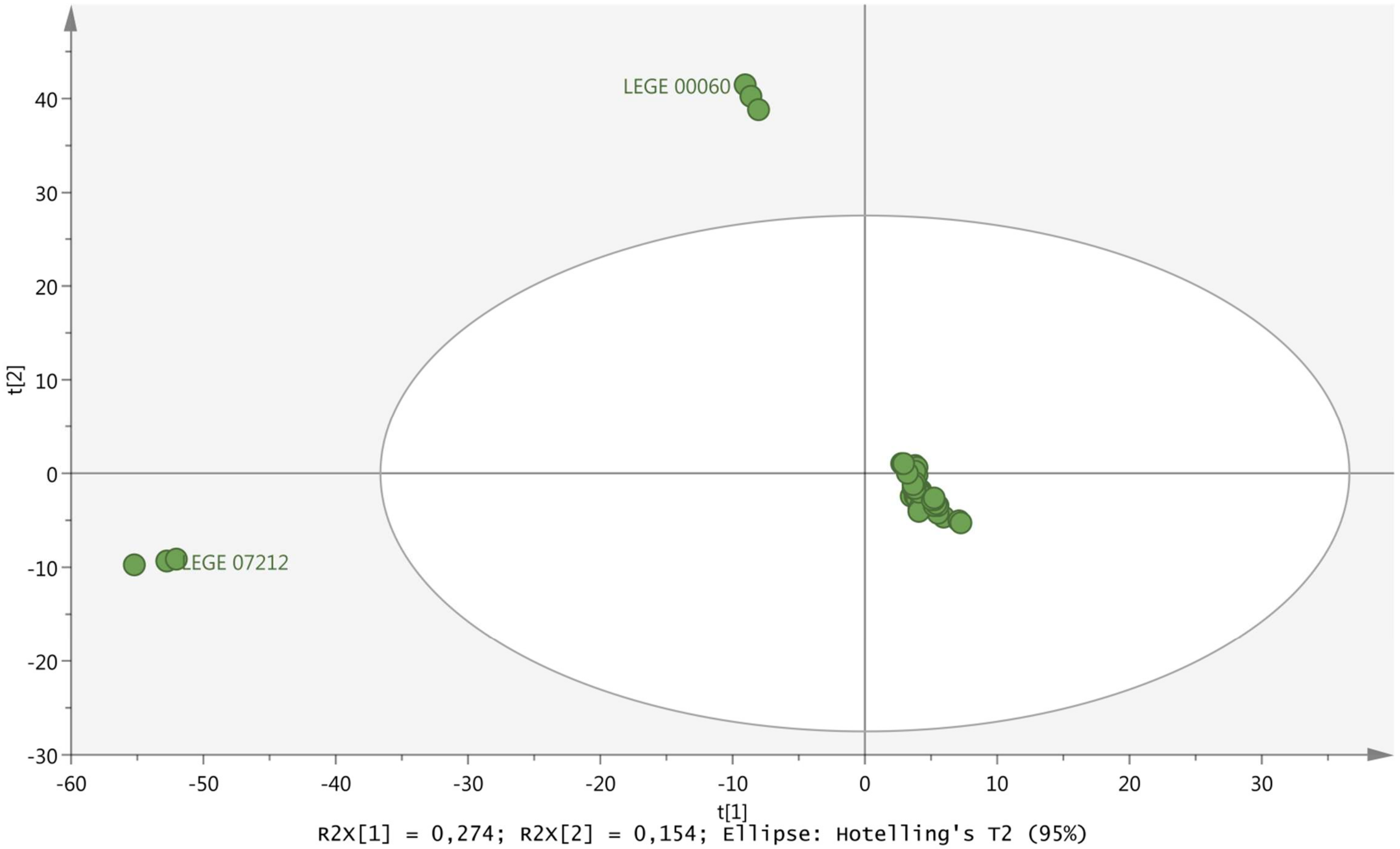

Figure 12 - Principal component analysis (scores plot) of cyanobacterial fractions I (VLC).

Supplementary Figure 13 - Representative bright-field images of brown adipocytes differentiated with different cyanobacterial fractions at 10 μg.mL^{-1}. The black arrows indicate the respective changes in mRNA expression of UCP-1 and PPARγ.

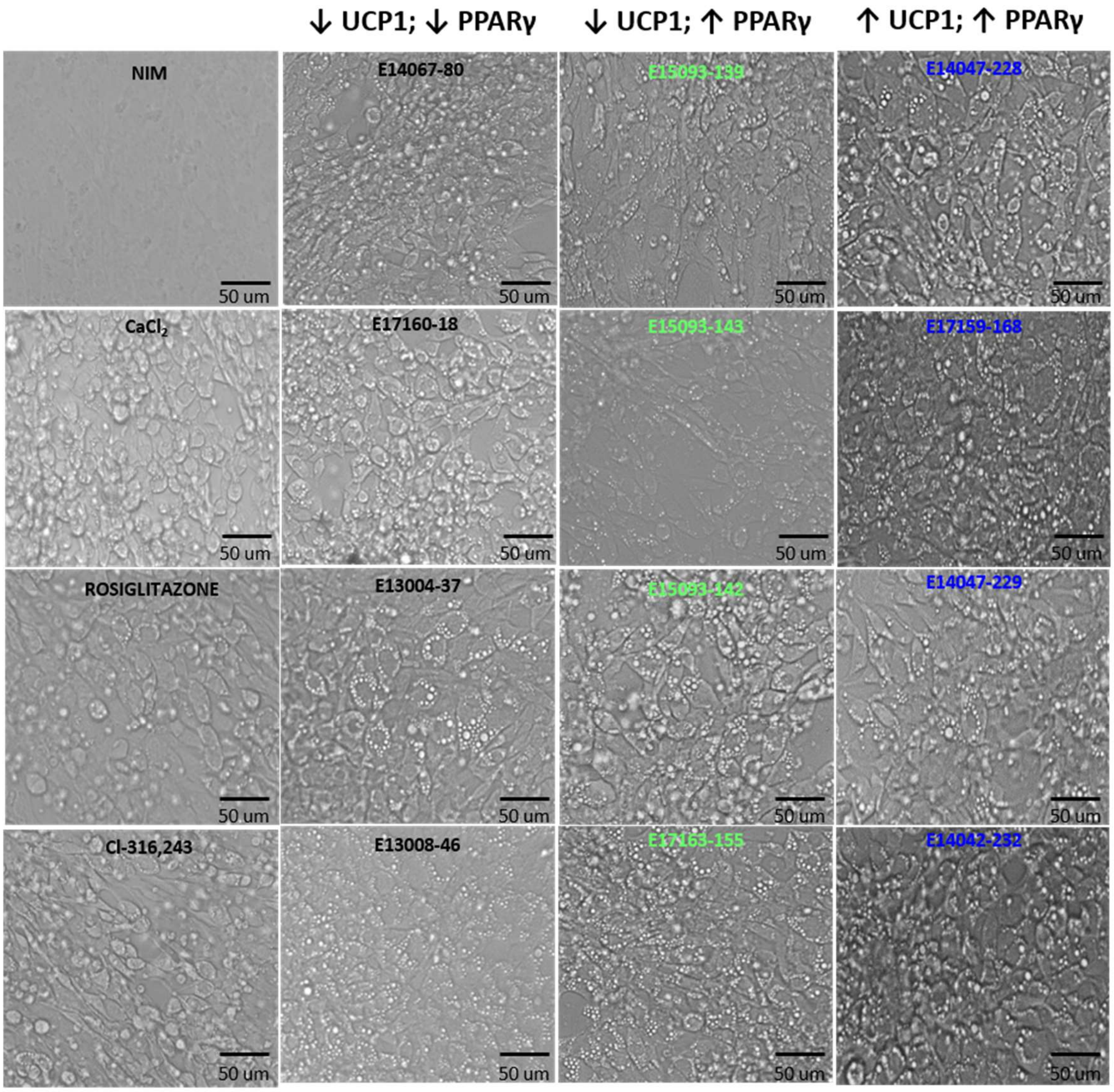

Table 3 – Identification of the compounds present in each fraction with activity in the zebrafish Nile red fat metabolism assay. RT retention time, m/z mass-to-charge ratio.

Fraction	RT	*m/z*	Adduct	Formula	Compound/Class
LEGE07175 H #134	5.62	338.3500			
	6.85	695.4724		$C_{32}H_{44}N_{14}O_3$	Could be new
	6.94	721.4817			
	7.60	723.5089			
	7.68	749.5248			
	8.25	725.5168			
LEGE00246 G #180	5.11	587.4642			
	5.95	791.5232			
	6.67	771.4998		$C_{39}H_{65}N_9O_7$	Peptide
	6.85	797.5310			
	7.18	747.5030			
	7.26	773.5234			
	7.88	775.5417			
LEGE07172 A #240	3.66	593.2729			
	4.89	607.2897	$[M+H]^+$	$C_{36}H_{38}N_4O_5$	Phaeophorbide A
	5.62	338.3441			
	7.30	553.3917	$[M+Na]^+$	$C_{37}H_{54}O_2$	Glycolipid
	10.42	871.5721	$[M+H]^+$	$C_{55}H_{74}N_4O_5$	Phaeophytin A
LEGE07172 C #242	6.86	885.5662			
	6.95	911.5776			
	7.61	723.5087			
	7.66	749.5176			
	8.27	751.5330			
LEGE 07173 B #256	4.34	585.4277	$[M+H]^+$	$C_{40}H_{56}O_3$	Dihydrodiadinoxanthin A<br>Antheraxanthin<br>Loroxanthin<br>Mutatoxanthin<br>Myxol<br>Pyrenoxanthin
	5.19	955.5428	$[M+Na]^+$	$C_{26}H_{54}N_{34}O_5$	Could be new
	5.44	569.4319	$[M+H]^+$	$C_{25}H_{52}N_{12}O_3$	Could be new
	6.26	388.3453			
	7.97	553.3994	$[M+Na]^+$	$C_{37}H_{54}O_2$	Glycolipid
	8.96	924.5458		$C_{51}H_{72}N_8O_8$	Could be new
	9.07	908.5311			
	9.38	663.4471	$[M+H]^+$	$C_{38}H_{62}O_9$	Eryloside T
	9.47	936.5570			

RT – retention time; *m/z* – mass to charge ratio

Table 4 - Identification of the compounds present in each fractions with activity in the anti-steatosis assay in HepG2 cells. RT retention time, m/z mass-to-charge ratio.

Fraction	RT	*m/z*	Adduct	Formula	Compound/Class
LEGE 07084 D #48	4.30	284.2963			
	5.22	955.5509	$[M+Na]^+$	$C_{26}H_{54}N_{34}O_5$	Could be new
	5.62	338.3460			
	7.31	797.5139			
	7.31	553.3862	$[M+Na]^+$	$C_{37}H_{54}O_2$	Glycolipid
	8.34	908.5446			
	10.17	887.5687	$[M+H]^+$	$C_{55}H_{74}N_4O_6$	Hydroxypheophytin A
				$C_{59}H_{74}N_4O_3$	Porphyrinolactone
LEGE 03283 C #108	5.62	338.3441			
	7.12	429.3765	$[M+ Na]^+$	$C_{54}H_{75}N_7O_4$	Could be new
	7.32	553.4000	$[M+Na]^+$	$C_{37}H_{54}O_2$	Glycolipid
	7.75	907.5519	$[M+Na]^+$		Could be new
	10.16	887.5646	$[M+H]^+$	$C_{55}H_{74}N_4O_6$	Hydroxypheophytin A
				$C_{59}H_{74}N_4O_3$	Porphyrinolactone
LEGE 03283 D #109	3.37	609.2731	$[M+H]^+$	$C_{35}H_{36}N_4O_6$	Phaeophorbide A
	5.07	955.5503	$[M+Na]^+$	$C_{26}H_{54}N_{34}O_5$	Could be new
	5.62	338.3464			
	7.30	553.3950	$[M+Na]^+$	$C_{37}H_{54}O_2$	Glycolipid
	8.32	908.5286			
	8.60	938.5466			
	10.14	887.5580	$[M+H]^+$	$C_{55}H_{74}N_4O_6$	Hydroxypheophytin A
				$C_{59}H_{74}N_4O_3$	Porphyrinolactone
	10.42	871.5729	$[M+H]^+$	$C_{55}H_{74}N_4O_5$	Phaeophytin A
LEGE 07167 B #199	1.67	439.0720	$[M+H]^+$		
	1.97	441.0870	$[M+H]^+$	$C_{28}H_{12}N_2O_4$	Could be new
	2.31	579.0629	$[M+Na]^+$	$C_{25}H_{24}N_4O_3S_4$	Leptosin F
	3.52	637.3085	$[M+H]^+$	$C_{37}H_{40}N_4O_6$	Phaeophytin analogue
	5.62	338.3441			
	6.84	797.5139			
	7.29	553.3917	$[M+Na]^+$	$C_{37}H_{54}O_2$	Glycolipid
	7.88	755.5498			
LEGE 07160 B #202	3.32	350.2514			
	4.95	569.4386			
	6.14	769.4828			
	6.72	771.4993			
	7.28	773.5289			
	7.31	553.3937	$[M+Na]^+$	$C_{37}H_{54}O_2$	Glycolipid
	7.82	775.5286			
	8.34	777.5444			
	8.73	685.4352	$[M+Na]^+$	$C_{38}H_{58}N_6O_4$	Could be new
	10.07	903.5668	$[M+H]^+$	$C_{50}H_{70}N_{12}O_4$	Could be new
	10.16	887.5709	$[M+H]^+$	$C_{55}H_{74}N_4O_6$	Hydroxypheophytin A

Fraction	RT	*m/z*	Adduct	Formula	Compound/Class
				$C_{59}H_{74}N_4O_3$	Porphyrinolactone
LEGE 06134 B #220	5.16	955.5492	[M+Na]+	$C_{26}H_{54}N_{34}O_5$	Could be new
	6.26	338.3412			
	7.98	553.3904	[M+Na]+	$C_{37}H_{54}O_2$	Glycolipid
	8.54	887.5818			
	9.10	871.5833			
	9.34	892.5394		$C_{55}H_{72}MgN_4O_5$	Chlorophyll a

RT – retention time; *m/z* – mass to charge ratio

Table 5 - Identification of the compounds present in each fraction with activity in the anti-diabetes 2-NBDG assay in HepG2 cells. RT retention time, m/z mass-to-charge ratio.

Fraction	RT	*m/z*	Adduct	Formula	Compound/Class
LEGE06001 G #25	5.62	338.3416			
	7.02	937.5682	$[M+H]^+$	$C_{41}H_{68}N_{20}O_6$	Could be new
	7.31	553.3962	$[M+Na]^+$	$C_{37}H_{54}O_2$	Glycolipid
	8.36	777.5515			
	8.77	663.4561	$[M+H]^+$	$C_{44}H_{58}N_2O_3$	Terpene
	10.08	903.5671	$[M+H]^+$	$C_{50}H_{70}N_{12}O_4$	Could be new
LEGE 06104 E #58	4.93	568.4274		$C_{40}H_{56}O_2$	Lutein
					Rubixanthin-5,6 epoxide
					Saproxanthin
					Tunaxanthin
					Zeaxanthin
					Cryptoflavin
					Isozeaxanthin
	5.62	338.3414			
	6.07	429.3904	$[M+ Na]^+$	$C_{54}H_{75}N_7O_4$	Could be new
	7.31	553.3962	$[M+Na]^+$	$C_{37}H_{54}O_2$	Glycolipid
	8.26	924.5337			
	10.15	887.5753	$[M+H]^+$	$C_{55}H_{74}N_4O_6$	Hydroxypheophytin A
				$C_{59}H_{74}N_4O_3$	Porphyrinolactone
LEGE 07172 C #77	3.66	593.2793			
	5.61	338.3395			
	7.31	553.3914	$[M+Na]^+$	$C_{37}H_{54}O_2$	Glycolipid
	10.06	903.5620	$[M+H]^+$	$C_{50}H_{70}N_{12}O_4$	Could be new
	10.16	887.5667	$[M+H]^+$	$C_{55}H_{74}N_4O_6$	Hydroxypheophytin A
				$C_{59}H_{74}N_4O_3$	Porphyrinolactone
	10.43	871.5721	$[M+H]^+$	$C_{55}H_{74}N_4O_5$	Phaeophytin A
LEGE 07175 E #130	5.03	955.5380	$[M+Na]^+$	$C_{26}H_{54}N_{34}O_5$	Could be new
	8.33	908.5272			
	8.50	892.5292		$C_{55}H_{72}MgN_4O_5$	Chlorophyll A

RT – retention time; *m/z* – mass to charge ratio

Table 6 - Identification of the compounds present in each fraction with activity in the PPARy and UCP1 assay in brown adipocytes. RT retention time, m/z mass-to-charge ratio.

Fraction	RT	*m/z*	Adduct	Formula	Compound/Class
LEGE 00247 D #168	5.62	338.3422			
	6.26	565.4067	$[M+H]^+$	$C_{40}H_{52}O_2$	Alloxanthin
					ε,ε-Carotene-3,3'-dione
					Eschscholtzxanthin
					Isozeaxanthin
	7.31	553.3876	$[M+Na]^+$	$C_{37}H_{54}O_2$	Glycolipid
	10.17	887.5697	$[M+H]^+$	$C_{55}H_{74}N_4O_6$	Hydroxypheophytin A
				$C_{59}H_{74}N_4O_3$	Porphyrinolactone
	10.44	871.5709	$[M+H]^+$	$C_{55}H_{74}N_4O_5$	Phaeophytin A
LEGE 06137 A #228	6.77	915.6031	$[M+H]^+$	$C_{49}H_{86}O_{15}$	Inulagalactolipid A
	7.29	553.3933	$[M+Na]^+$	$C_{37}H_{54}O_2$	Glycolipid
	10.13	887.5688	$[M+H]^+$	$C_{55}H_{74}N_4O_6$	Hydroxypheophytin A
				$C_{59}H_{74}N_4O_3$	Porphyrinolactone
	10.43	871.5649	$[M+H]^+$	$C_{40}H_{70}N_{16}O_6$	Could be new
LEGE 06097 B #232	3.91	585.4333	$[M+H]^+$	$C_{40}H_{56}O_3$	Dihydrodiadinoxanthin A
					Antheraxanthin
					Loroxanthin
					Mutatoxanthin
					Myxol
					Pyrenoxanthin
	4.98	955.5469	$[M+Na]^+$	$C_{26}H_{54}N_{34}O_5$	Could be new
	5.62	338.3427			
	7.30	553.3942			
	7.62	723.4991			
	8.29	887.5785			
	10.17	887.5807	$[M+H]^+$	$C_{55}H_{74}N_4O_6$	Hydroxypheophytin A
				$C_{59}H_{74}N_4O_3$	Porphyrinolactone

RT – retention time; *m/z* – mass to charge ratio
